# BAG3 promotes pancreatic ductal adenocarcinoma growth by activating stromal macrophages

**DOI:** 10.1038/ncomms9695

**Published:** 2015-11-02

**Authors:** Alessandra Rosati, Anna Basile, Raffaella D'Auria, Morena d'Avenia, Margot De Marco, Antonia Falco, Michelina Festa, Luana Guerriero, Vittoria Iorio, Roberto Parente, Maria Pascale, Liberato Marzullo, Renato Franco, Claudio Arra, Antonio Barbieri, Domenica Rea, Giulio Menichini, Michael Hahne, Maarten Bijlsma, Daniela Barcaroli, Gianluca Sala, Fabio Francesco di Mola, Pierluigi di Sebastiano, Jelena Todoric, Laura Antonucci, Vincent Corvest, Anass Jawhari, Matthew A Firpo, David A Tuveson, Mario Capunzo, Michael Karin, Vincenzo De Laurenzi, Maria Caterina Turco

**Affiliations:** 1 BIOUNIVERSA s.r.l., Fisciano, Salerno 84084, Italy; 2 Department of Medicine and Surgery, University of Salerno, Baronissi, Salerno 84081, Italy; 3 Department of Pharmacy, Division of Biomedicine “A. Leone”, University of Salerno, Fisciano, Salerno 84084, Italy; 4 Pathology Unit, Istituto Nazionale Tumouri Fondazione “G. Pascale”, Naples 81100, Italy; 5 Animal facility, Istituto Nazionale Tumouri Fondazione “G. Pascale”, Naples 81100, Italy; 6 Reconstructive Microsurgery, Department of Oncology, Careggi University Hospital, Florence 50139, Italy; 7 Institut de Génétique Moléculaire de Montpellier, CNRS UMR5535, Montpellier 34293, France; 8 Laboratory for Experimental Oncology and Radiobiology, Academic Medical Center, University of Amsterdam, Amsterdam 1105AZ, The Netherlands; 9 Dipartimento di Scienze Mediche, Orali e Biotecnologiche, University “G. d'Annunzio” di Chieti-Pescara, Centro Studi sull'Invecchiamento, CeSI-MeT, Chieti 66100, Italy; 10 Division of Surgical Oncology, “S.S. Annunziata”Hospital, Chieti 66100, Italy; 11 Laboratory of Gene Regulation and Signal Transduction, Departments of Pharmacology and Pathology, UCSD, School of Medicine, San Diego, California 92093-0723, USA; 12 CALIXAR, Bioparc, Bâtiment Laënnec, Lyon 69008, France; 13 Department of Surgery, Huntsman Cancer Institute, University of Utah School of Medicine, Salt Lake City, Utah 84132, USA; 14 Cold Spring Harbor Laboratory, Cold Spring Harbor, New York 11724, USA

## Abstract

The incidence and death rate of pancreatic ductal adenocarcinoma (PDAC) have increased in recent years, therefore the identification of novel targets for treatment is extremely important. Interactions between cancer and stromal cells are critically involved in tumour formation and development of metastasis. Here we report that PDAC cells secrete BAG3, which binds and activates macrophages, inducing their activation and the secretion of PDAC supporting factors. We also identify IFITM-2 as a BAG3 receptor and show that it signals through PI3K and the p38 MAPK pathways. Finally, we show that the use of an anti-BAG3 antibody results in reduced tumour growth and prevents metastasis formation in three different mouse models. In conclusion, we identify a paracrine loop involved in PDAC growth and metastatic spreading, and show that an anti-BAG3 antibody has therapeutic potential.

## Abstract (web summary)

BAG3 is found in the serum of pancreatic cancer patients and can be used as a marker of disease, but its role in cancer is unclear. Here, the authors show that BAG3 secreted from tumour cells binds to and activates macrophages, which in turn promotes cell growth, and an antibody blocking BAG3 binding reduces tumour formation in mice.

## Graphical abstract

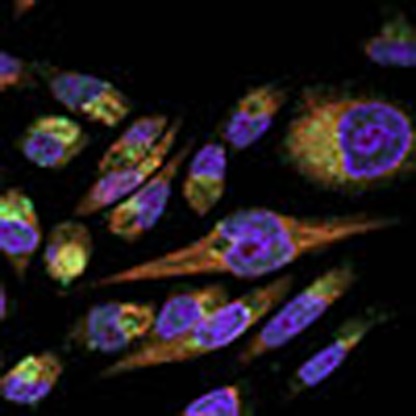

Pancreatic ductal adenocarcinoma (PDAC) is the only one of the five most lethal malignancies for which both the incidence and death rate have increased in recent years^1^,^2^. This is due to the fact that it is frequently detected only in an advanced stage and to its resistance to current therapies; therefore the identification of novel targets for treatment is of great clinical importance. Of particular interest is the identification of molecules that mediate the interaction between the tumour and the surrounding stroma, including tumour-infiltrating macrophages, which are critically involved in pancreatic tumour formation, progression and metastatization^3^,^4^,^5^,^6^,^7^,^8^,^9^,^10^,^11^,^12^,^13^,^14^,^15^,^16^.

We have previously reported the intracellular expression of BAG3 (*Bcl-2-*associated athanogene 3) in 346/346 PDAC biopsies and its correlation with prognosis^17^. BAG3 belongs to a family of co-chaperones that interact with the ATPase domain of the heat shock protein (Hsp) 70 through a structural domain known as the BAG domain^18^,^19^. *Bag3* gene expression is constitutive only in a few normal cell types, including skeletal muscle and cardiac myocytes, while can be induced by different types of stress in many other cell types. Interestingly, BAG3 is constitutively expressed in several primary tumours or tumour cell lines, where it has been shown to exert a pro-survival role through various mechanisms that vary according to cellular context^20^,^21^,^22^. Recently, we reported that BAG3 is also detectable in serum samples from PDAC patients^23^, suggesting a role for secreted BAG3 in tumour development. Here we show that indeed BAG3 is released by PDAC cells and activates macrophages through a specific receptor, IFITM-2 (Interferon-Induced Transmembrane Protein 2). BAG3-activated macrophages secrete factors that stimulate PDAC cell proliferation. Interruption of this loop through an anti-BAG3 monoclonal antibody impairs tumour growth and metastasis formation.

## Results

### BAG3 is released from PDAC cells and activates macrophages

**Figure 1:**
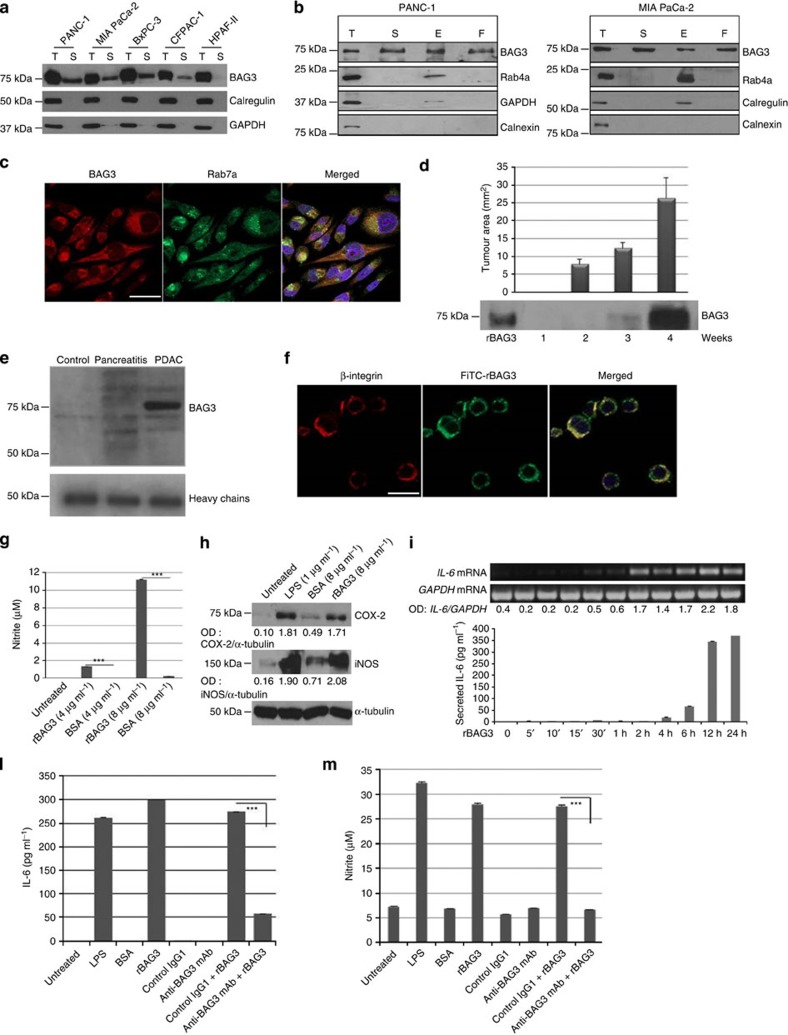
BAG3 is released from PDAC cells and activates macrophages. (**a**) PDAC total proteins (T) and proteins from supernatants (S) were analysed by western blotting (WB) using an anti-BAG3 pAb. Anti-calnexin and anti-GAPDH were used as controls. (**b**) PDAC proteins: total (T), from supernatants (S), from exosomes (E), extracellular (not associated to exosomes) (F), were analysed by WB using an anti-BAG3 pAb. Anti-Rab-4a was used as exosomes marker. Anti-calnexin, anti-calregulin and anti-GAPDH were used as controls. (**c**) MIA PaCa-2 was analysed for BAG3 co-localization with Rab7a by immunofluorescence; overlap coefficient (ImageJ software) was 0.8 (scale bar, 20 μm). (**d**) MIA PaCa-2 was transplanted in the pancreas of nude mice. The graph depicts mean (±s.e.m.) of tumour areas (measured by ultrasound imaging) at indicated times in three animals. Serum levels of BAG3 were analysed from sera pooled from the three animals by WB using an anti-BAG3 mAb. rBAG3 was loaded as a control. (**e**) Sera from normal pancreas, chronic pancreatitis and PDAC-carrying mice were immune-precipitated with an anti-BAG3 mAb. BAG3 was assessed by WB using the anti-BAG3 pAb. (**f**) J774A.1 was incubated with FITC-conjugated rBAG3 and analysed by confocal microscopy. A rhodamine-conjugated anti-β-integrin mAb was used as plasma membrane marker (scale bar, 20 μm). (**g**) J774A.1 was incubated for 24 h with rBAG3 or BSA; nitrite release was measured in supernatants using Griess reagent. Data are from duplicate samples and confirmed in two separate experiments. Error bars indicate s.d. (**h**) J774A.1 was treated for 16 h with LPS, rBAG3 or BSA. Protein extracts were analysed by WB using the indicated antibodies. α-tubulin antibody was used as loading control. (**i**) J774.A1 was treated with rBAG3 for the indicated times; then cell total RNA was extracted and IL-6 evaluated in the supernatants by ELISA test. Data are from triplicate samples and repeated two times. Error bars indicate s.d. (**l**) J774A.1 cells were incubated with LPS, rBAG3 or BSA for 24 h in the absence or presence of an anti-BAG3 mAb (320 μg ml^−1^). Murine IgG1 were used as negative control. Supernatants were analysed with a mouse IL-6 ELISA Kit. Data are from duplicate samples and confirmed in two separate experiments. Error bars indicate s.d. (**m**) Cells were treated as described above and nitrite release measured as described in **g**. Data are from duplicate samples and confirmed in two separate experiments. Error bars indicate s.d. *P* values were calculated by Student's *t*-test: ***P<0.001.

We initially investigated extracellular release of BAG3 from five different human PDAC cell lines. All the tested cell lines expressed intracellular BAG3 protein and appeared to release it in the culture supernatant (Fig. 1a). Differential centrifugation of subcellular fractions shows that BAG3 is detectable in both the exosome and soluble fractions of PANC-1 and MIA PaCa-2 cell lines (Fig. 1b). BAG3 also co-localizes with Rab7a, a cytosolic marker for endosomes, suggesting that it might be secreted through the exosomal pathway (Fig. 1c)^24^. BAG3 release was also detectable in serum samples obtained from (nu/nu) mice orthotopically xenografted with MIA PaCa-2 cells (Fig. 1d). Importantly, BAG3 serum amounts appeared to correlate with tumour size. Moreover BAG3 secretion does not appear to be a specific feature of human PDAC cell lines, as we could detect BAG3 in sera from Pdx-Cre; KrasG12D, Ikkalpha f/f mice^25^ that spontaneously develop PDAC, while it was undetectable in Pdx-Cre, Ikkalpha f/f mice that only develop pancreatitis (Fig. 1e).

We first hypothesized that released BAG3 could act as an autocrine factor, however, we could not detect binding of fluorescein isothiocyanate (FITC)-conjugated recombinant (r) BAG3 to the surface of PDAC cell lines (Supplementary Fig. 1A). Nevertheless, we detected binding of BAG3 to the surface of the murine macrophage cells J774.A1 and human monocytes (Fig. 1f and Supplementary Fig. 1B). We therefore tested if BAG3 was capable of activating J774.A1 cells. On incubation with rBAG3, J774.A1 cells release nitrites and express higher amounts of COX-2 (Cyclooxygenase-2) and iNOS (Nitric Oxide Synthase-2, Inducible) revealing macrophage activation (Fig. 1g,h). Moreover, J774.A1 cells incubated with BAG3 showed increased IL-6 (Interleukin-6) mRNA levels and IL-6 secretion (Fig. 1i). Notably, IL-6 is one of the main factors released in the PDAC microenvironment and known to play a role in tumour development^26^,^27^,^28^. Importantly, a monoclonal anti-BAG3 antibody, that we generated, inhibited the binding of BAG3 to the surface of J774.A1 cells (Supplementary Fig. 1C) and consequently blocked their activation (Fig. 1l,m), while, as expected, it had no effect on lipopolysaccharide (LPS)-dependent activation (Supplementary Fig. 1D). Furthermore, non-specific macrophage activation due to potential LPS contamination of rBAG3 preparations can be excluded, since pre-treatment with polymyxin B did not affect BAG3-dependent IL-6 release (Supplementary Fig. 1D).

**Figure 2:**
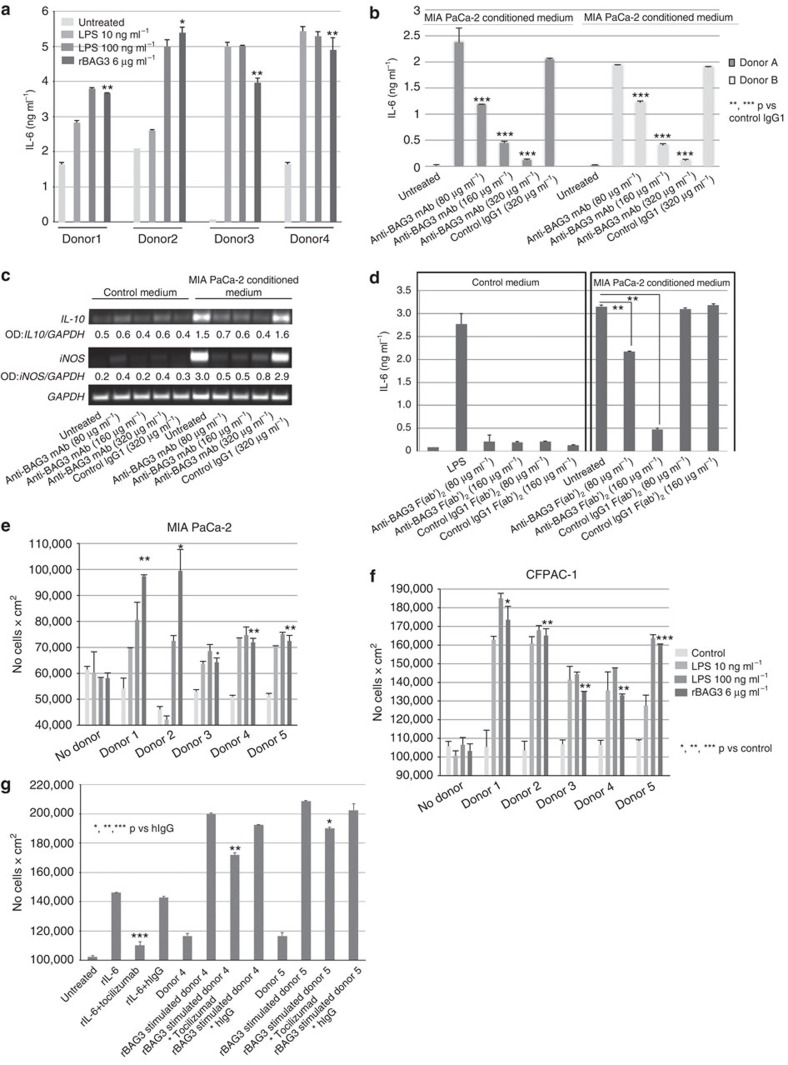
PDAC cell supernatants activate macrophages that in turn secrete molecules that promote PDAC cells proliferation. (**a**) Isolated human monocytes (>98% CD14^+^) were treated with LPS or rBAG3 for 16 h. Then, supernatants were analysed with human IL-6 ELISA. Data are from duplicate samples and obtained in two separate experiments. Error bars indicate s.d. (**b**) Isolated human monocytes (>98% CD14^+^) were cultured in RPMI supplemented with 0.15% FBS (control medium) or in MIA PaCa-2-conditioned medium and treated for 16 h with an anti-BAG3 monoclonal antibody at the indicated concentrations. Unrelated murine IgG1 were used as negative control. After treatment, supernatants were collected and analysed with a human IL-6 ELISA Kit. Data are from triplicate samples and obtained in two separate experiments. Error bars indicate s.d. (**c**) Cells were treated as described above. Cells were then harvested for total RNA extraction and analysed by reverse transcription–PCR (RT–PCR). (**d**) Isolated human monocytes were cultured in RPMI supplemented with 0.15% FBS (control medium) or in MIA PaCa-2-conditioned medium, and treated with the F(ab')_2_ fragment of an anti-BAG3 monoclonal antibody. F(ab')_2_ fragments of a non-specific murine IgG1 were used as negative control. After treatment, supernatants were collected and analysed with a human IL-6 ELISA Kit. Data are from duplicate samples and repeated two times. Error bars indicate s.d. (**e**) MIA PaCa-2 and CFPAC-1 (**f**) cells were cultured in DMEM supplemented with 0.15% FBS (no donor) or conditioned medium from monocytes treated with LPS or rBAG3 for 16 h at the indicated concentrations. After 72 h incubation, cells were analysed by MTT assay. Data are from duplicate samples. Error bars indicate s.d. (**g**) MIA PaCa-2 cells were incubated with human recombinant (**r**) IL-6 (10 ng ml^−1^) or conditioned medium from donor 4 or donor 5 monocytes stimulated with rBAG3 (6 μg ml^−1^) for 16 h. An anti-IL-6 receptor monoclonal antibody (20 μg ml^−1^) was added to inhibit IL-6 activity. After a 72-h incubation, cells were analysed by MTT assay. Data are from duplicate samples. Error bars indicate s.d. *P* values were calculated by Student's *t*-test and represented as follows: *P<0.05>0.01; **P<0.01>0.001; ***P<0.001.

These results were confirmed using primary monocytes from the peripheral blood of healthy donors. Purified (>98% CD14^+^) monocytes from four different donors appeared to release IL-6 on incubation with rBAG3 (Fig. 2a). Moreover, we found that conditioned medium from PDAC cells was effective in activating human peripheral blood monocytes, as indicated by IL-6 release, and that the addition of anti-BAG3 mAb to cell cultures abrogated this effect on IL-6 production (Fig. 2b) and on induction of IL-10 and iNOS (Fig. 2c). Similar results were obtained using an F(ab')_2_ fragment of the antibody (Fig. 2d), further confirming antibody specificity.

### Macrophages release factors that promote PDAC cell growth

We assumed that BAG3-activated macrophages might release factors that sustain PDAC tumour growth and metastasis formation. Indeed, we found that proliferation of two different human PDAC cell lines, MIA PaCa-2 and CFPAC-1, was enhanced by addition of conditioned medium from BAG3-activated monocytes cultures (Fig. 2e,f), but not by treatment with rBAG3 (Fig. 2e,f no donor). As BAG3 does not function in an autocrine manner, anti-BAG3 mAb treatment of those cell lines did not alter their growth (Supplementary Fig. 2A,B). The stimulatory effect of the supernatants was not proportional to their IL-6 content (Table 1), indicating that it was not attributable exclusively to the effect of this cytokine. This was confirmed by the fact that an anti-IL-6 receptor mAb (Toclizumab), while antagonizing >80% the effect of rIL-6 (10 ng ml^−1^) on MIA PaCa-2 cell proliferation, inhibited only partially (<25%) the effect of the conditioned medium from BAG3- stimulated monocytes (Fig. 2g and Table 2).

### Identification of BAG3 receptor

**Figure 3:**
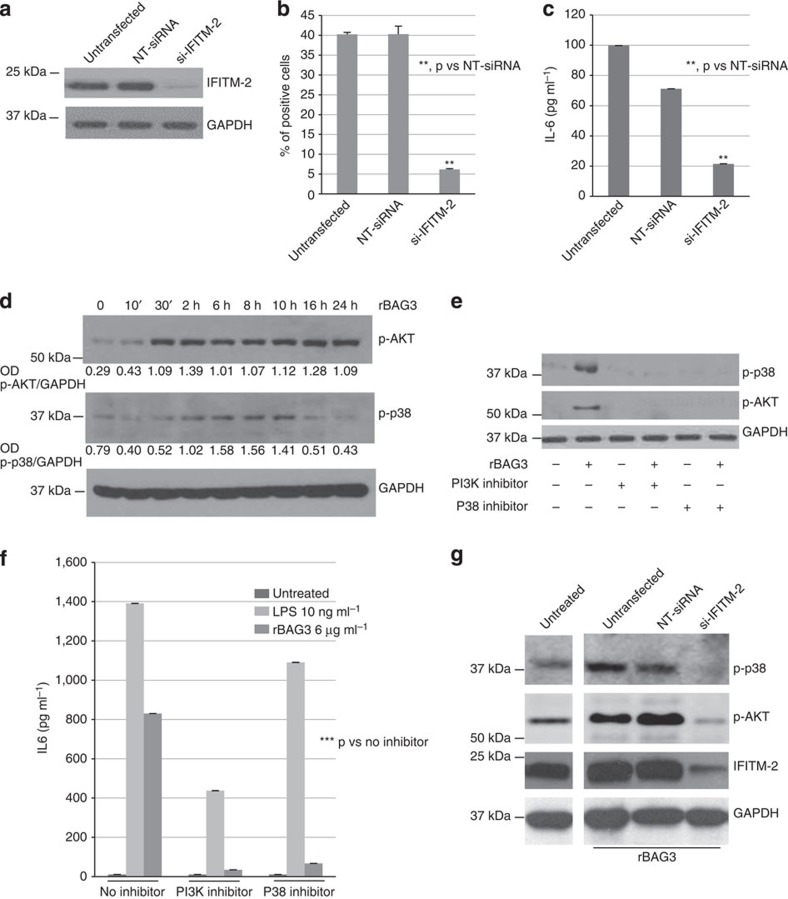
IFTM-2 acts as a BAG3 receptor. (**a**) J774.A1 were plated in 24-well plates at 40% confluence. After 24 h, cells were transfected with an IFITM-2-specific siRNA (si-IFITM-2); a non-targeted (NT) siRNA was used as a control. After 48 h, cells were harvested and total cell extracts were analysed by WB with an anti-IFITM-2 polyclonal antibody; an anti-GAPDH antibody was used as a loading control. (**b**) J774.A1 were transfected as described above. After 48 h, cells were harvested, stained with FITC-conjugated rBAG3 and analysed by flow cytometry. Data are from triplicate samples and confirmed in three separate experiments. Error bars indicate s.d. (**c**) J774.A1 were transfected as described above. After 16 h of stimulation with rBAG3, IL-6 content was analysed in supernatants by ELISA. Data are from triplicate samples and confirmed in three separate experiments. Error bars indicate s.d. (**d**) J774.A1 cells were treated with rBAG3 (6 μg ml^−1^) and cells harvested at the indicated time points. Cell extracts were analysed by western blotting using anti-phospho-AKT and anti-phospho-p38 polyclonal antibodies; anti-GAPDH was used as a loading control. (**e**) J774.A1 cells were incubated for 30 min with the PI3K (LY294002) or p38 (SB203580) inhibitors, then rBAG3 (6 μg ml^−1^) was added for additional 8 h to the cultures. Proteins were analysed by WB using anti-phospho-AKT and anti-phospho-p38 polyclonal antibodies; anti-GAPDH was used as a loading control. (**f**) J774.A1 cells were incubated for 30 min with the PI3K (LY294002) or p38 (SB203580) inhibitors, then rBAG3 was added for additional 16 h to the cultures. IL-6 content in supernatants was analysed by ELISA. Data are from duplicate samples and repeated three times. Error bars indicate s.d. (**g**) J774.A1 were transfected with si-IFITM-2, a non-targeted (NT) siRNA was used as a control. Cells were incubated with rBAG3 (6 μg ml^−1^) for 16 h, then total protein extracts were analysed by WB with the indicated antibodies. *P* values were calculated by Student's *t*-test and represented as follows: **P<0.01>0.001; ***P<0.001.

Since BAG3 binds to the surface of monocytes/macrophages and activates them, we aimed to identify the cell surface receptor for BAG3. For this, J774 macrophages were incubated with His-tagged recombinant BAG3 and subjected to plasma membrane fractionation, sequential centrifugation, identification of BAG3-containing fractions by western blotting, extraction of membrane proteins using detergents and finally isolation of BAG3-containing complexes by affinity chromatography on nickel-charged resin (Supplementary Fig. 3A,B). Liquid chromatography–tandem mass spectrometry (LC–MS/MS) analysis of BAG3-co-purified proteins identified a small number of proteins. Among these, IFITM-2 (Supplementary Fig. 3C and Supplementary Table 1) was the only transmembrane protein and therefore a potential candidate as a cell surface receptor for BAG3. The binding between BAG3 and IFITM-2 was also confirmed by co-immunoprecipitation (Supplementary Fig. 3C). To confirm the role of IFTM-2 as BAG3 receptor, we analysed BAG3 binding and cell activation on IFITM-2-silenced J774.A1 cells (Fig. 3a). Indeed, binding of FITC-rBAG3 (Fig. 3b) and release of IL-6 (Fig. 3c) were both inhibited by IFITM-2 silencing by >75%. We then investigated the potential signalling pathways downstream of BAG3 binding to IFITM-2 focusing on the PI3K (phosphatidylinositol 3-kinase) and the p38 MAPK pathways known to be involved in COX-2, iNOS and IL-6 induction in macrophages^29^,^30^,^31^. As shown in Fig. 3d, treatment with rBAG3 results in phosphorylation of AKT and p38. Phosphorylation of AKT was detectable earlier than p38 phosphorylation and more persistent. Furthermore the PI3K inhibitor LY294002 and the p38 inhibitor SB203580 effectively inhibited AKT and p38 phosphorylation (Fig. 3e), as well as IL-6 release (Fig. 3f). As shown in Fig. 3g, we confirmed that BAG3 signalling is mediated by IFITM-2 since its silencing abrogated BAG3-induced phosphorylation of AKT and p38.

Since it is well-known that BAG3 binds to and cooperates with Hsp70 (ref. 18), it is possible that the effects we observed are also dependent on Hsp70 (ref. 19). However using a BAG3 mutant (R480A)^32^ that no longer binds to Hsp70 or YM-1, a specific inhibitor of the interaction between BAG3 and Hsp70 (ref. 33), we were able to demonstrate that indeed BAG3 binding to macrophages and their consequent activation do not require the interaction between BAG3 and Hsp70 (Supplementary Figs 4 and 5).

### Effect of BAG3 blocking on tumour growth and metastasis

**Figure 4:**
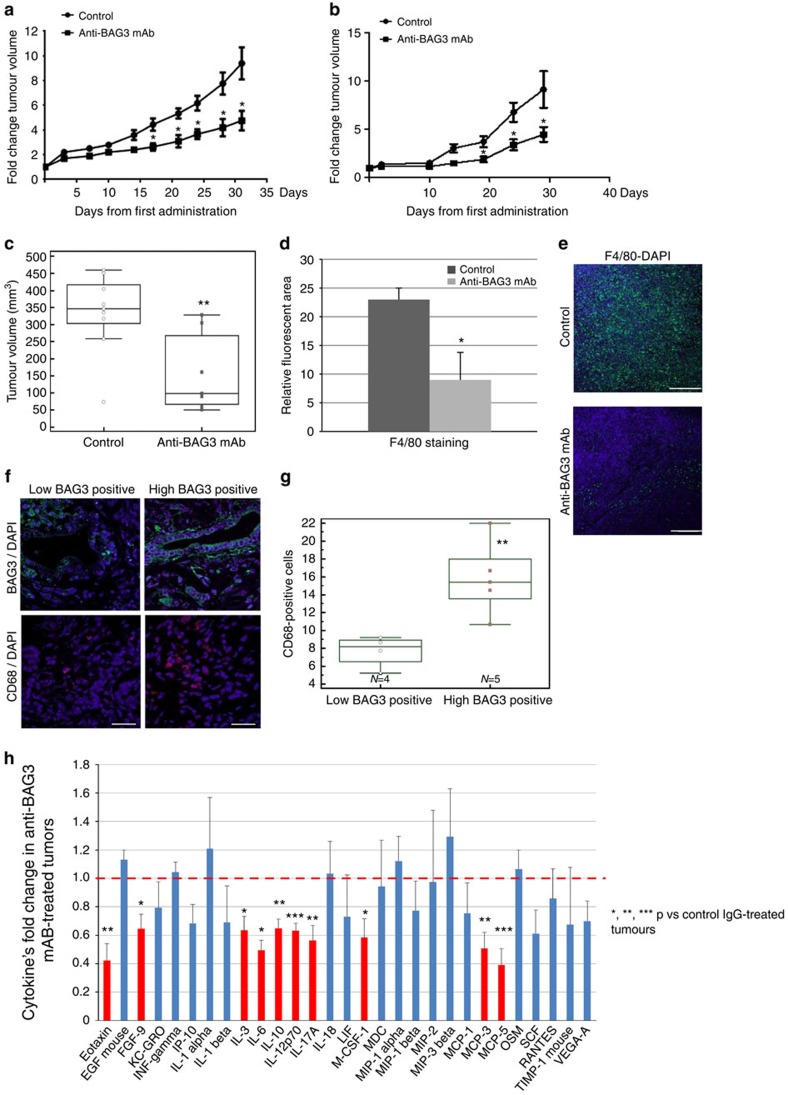
Anti-BAG3 mAb affects PDAC tumour growth and metastatic spreading. (**a**) *In vivo* response of a PDX tumour to treatment with vehicle (PBS) or anti-BAG3 mAb. Tumour volume was assessed by caliper. Results are expressed as mean fold change (±s.e.m.). (**b**) *In vivo* response of a syngeneic tumour (mt4-2D murine cells injected into C57BL6 mice) to treatment with control unrelated IgGs or anti-BAG3 mAb. Tumour volume was assessed by caliper. Results are expressed as mean fold change (±s.e.m.). (**c**) MIA PaCa-2 were transplanted orthotopically in the pancreas of nude mice. After tumour establishment, monitored by ultrasound imaging, animals were treated with control unrelated IgGs or anti-BAG3 mAb. Box-and-whisker graph depicts tumour volumes measured using an automated caliper *ex vivo* at the end of the experiment. The horizontal line represents the mean while whiskers s.d. (**d**) Tumour specimens were analysed by immunofluorescence using anti-F4/80. Relative fluorescence area of F4/80-positive cells was calculated as ratio to DAPI staining using ImageJ software from at least three images from × 10 field magnification. Error bars indicate s.d. (**e**) Representative images from the experiment described above (scale bar, 50 μm). (**f**) Samples from nine patients with stage 3 PDAC were stained with BAG3 and CD68. High BAG3 positivity was assigned when >50% of neoplastic cells were found positive, while with less we assigned a low positivity. Representative images from the two groups are shown (scale bar, 5 μm). (**g**) Box-and-whisker graph showing the number of macrophages into the two groups obtained by counting CD68-positive cells in at least five fields per patient sample. The horizontal line represents the mean while whiskers s.d. (**h**) Tumours from 6 anti-BAG3 mAb-treated mice and 5 control IgG-treated mice were analysed for cytokine contents using Myriad RBM Mouse Inflammation MAP v. 1.0 array. The graph depicts cytokines' fold change of anti-BAG3 mAb- with respect to IgG-treated tumours (±s.e.m.). The red dashed line represents the mean concentration of each cytokine in IgG-treated samples that was set equal to 1. Some cytokines from the panel were excluded since undetectable in our samples. *P* values were calculated by Student's *t*-test and represented as following: *P<0.05>0.01; **P<0.01>0.001; ***P<0.001.

Finally, we tested if neutralization of PDAC-released BAG3 by an anti- BAG3 mAb also impairs tumour growth and metastatic spreading *in vivo*. To this end, we established a patient-derived xenograft (PDX) model of PDAC. As shown in Fig. 4a (and Supplementary Fig. 6A), mice transplanted with the PDX, treated with the anti-BAG3 mAb administered every 48 h, showed a significant delay of the growth of xenografted tumours compared with the control group. Similar results were obtained while treating with the anti-BAG3 mAb, a graft obtained by s.c. injection of a murine Kras-driven pancreatic cancer cell line (mt4-2D)^34^ in C57BL6 syngeneic mice (Fig. 4b and Supplementary Fig. 6B), showing that the antibody is effective also in the presence of a fully developed immune system. We next investigated the effect of anti-BAG3 mAb treatment with the same dosage and administration schedule described above in an orthotopic PDAC model. In this model, MIA PaCa-2 cells were grafted into the pancreas of nude mice. This model has the advantage of resulting in the metastatic spreading of the primary tumour. In this model, treatment with anti-BAG3, but not with a control IgG, resulted in highly (>70%) reduced tumour volumes (Fig. 4c) and in complete prevention of metastatic spreading (Table 3). The anti-BAG3 mAb we used in our experiments appears to bind also murine BAG3 as shown by immunoprecipitation and staining of mouse heart tissue (Supplementary Fig. 6C,D). Importantly, no loss of weight (data not shown) or anatomical changes in main organs (Supplementary Fig. 6E) was observed in the group of animals treated with the anti-BAG3 mAb, excluding unwanted toxic effects. In accordance with our proposed mechanism-of-action, a comparison of tumour biopsies from anti-BAG3 mAb-treated or control animals showed a significant reduction in the number of stromal macrophages in the BAG3 mAb-treated group (Fig. 4d,e), confirming the capacity of the mAb to modulate monocyte/macrophage activation and infiltration of tumour stroma. Furthermore, analysis of macrophage infiltration in nine patients with stage 3 PDAC indicates a significant correlation between BAG3 expression and the number of infiltrating macrophages (Fig. 4f,g). Finally the impact of depleting secreted BAG3 in PDAC tumours is also reflected in a general decrease of macrophage-released cytokines (Fig. 4h).

## Discussion

It is well-known that inflammation plays a pivotal role in tumour initiation, promotion, development and metastasis^35^, in part through the action of secreted cytokines. This link between inflammation and cancer has extensively been shown also for PDAC. Indeed, PDAC growth and metastatic spreading appears to require the activity of a number of factors expressed in the tumour stroma, such as TGF-β, IL-6, CTGF (Connective Tissue Growth Factor), midkine, IGF-1 (Insulin-Like Growth Factor 1), IL-17 and others^26^,^27^,^28^,^36^,^37^,^38^,^39^,^40^,^41^,^42^,^43^,^44^; moreover these in turn activate stromal and infiltrating cells^26^,^27^,^28^,^42^,^43^,^44^. We have now identified a novel BAG3-mediated paracrine loop involving activation of macrophages and possibly other micro-environmental cells that support PDAC development and metastasis formation. Our *in vivo* experiments strongly support the tumour promoting effect of circulating BAG3 via modulating macrophage responses. The results obtained with the use of a syngeneic mice model suggests that this role is also exerted in the presence of a fully developed immune system. Additional studies, however, are required to fully dissect the effect of BAG3 in immune-competent mice. Though immune cells can execute anti-tumour responses, they are frequently educated by the tumour cells to become immune suppressors (for example, myeloid-derived suppressor cells and regulatory T cells). It is tempting to speculate an implication of BAG3 in the immune escape of pancreatic tumour cells.

We here report that through the release of BAG3, PDAC cells stimulate macrophage activation and the release of cancer cell-sustaining factors. Analogous paracrine interactions were described for other tumours, for instance, Lewis Lung Carcinoma cells secrete a proteoglycan, versican, that activates macrophages through Toll like receptors^45^. Similarly on irradiation, melanoma cells release soluble factors that induce an inflammatory response that promotes tumour regrowth^46^.

Notably, we could identify the receptor that mediates BAG3 activation of macrophages: IFITM-2 that belongs to a recently discovered family of proteins^47^. IFITM-2 is required for BAG3 binding and signalling, as its silencing results in almost complete abrogation of BAG3 binding to the macrophage surface and their activation via AKT and p38 phosphorylation. Importantly blocking this paracrine loop with an anti-BAG3 antibody results in reduced tumour growth and metastatic spreading. Our results suggest that this pathway is a potential target for designing novel therapeutic approaches against this deadly disease. Moreover since several other cancer types express intracellular BAG3 (refs 20, 21), they might also release it to the extracellular environment, extending the potential of the therapeutic use of BAG3-blocking antibody. Finally the fact that we are interfering with secreted BAG3 and not the intracellular one suggests that this approach should not be toxic for those tissues such as heart and muscle in which this protein is essential. This is confirmed by our preliminary results in mice.

## Methods

### Cell cultures

The murine macrophage cell line J774.A1 was purchased from the American Type Culture Collection (ATCC, Manassas, VA, USA) and cells were cultured in Dulbecco's Modified Eagle's Medium (DMEM) supplemented with 10% heat-inactivated foetal bovine serum (GIBCO, Life Technologies, Grand Island, NY, USA), penicillin and streptomycin (P/S, 100 U ml^−1^) (Lonza, Walkersville, MD, USA), L-glutamine (2 mM) (Lonza) and sodium pyruvate (1 mM) (EuroClone, MI, Italy). The human pancreatic cancer cell lines BxPC3, CFPAC-1, HPAF-2, PANC-1 and MIA PaCa-2 were obtained from ATCC. On receipt, each cell line was expanded, cryopreserved as low-passage stocks and tested routinely for mycoplasma immediately before use in an experiment. BxPC3 cell lines were cultured in RPMI-1640 (EuroClone) medium supplemented with 10% FBS and 1% P/S. HPAF-II were cultured in Eagle's Minimum Essential Medium (Lonza) supplemented with 10% FBS and 1% P/S. CFPAC-1 cell lines were cultured in Iscove's Modified Dulbecco's Medium (Gibco). PANC-1 and MIA PaCa-2 were cultured in DMEM medium containing 10% FBS and 1% P/S; 2.5% of horse serum (Gibco) was added in MIA PaCa-2 culturing medium. Human peripheral blood mononuclear cells were isolated by Lymphocyte Separation Medium (17-829F, Lonza) density gradient centrifugation. Monocytes (>98% CD14^+^) were isolated using the Monocyte Isolation Kit II (Miltenyi Biotec, Miltenyi Biotec S.r.l., BO, Italy) according to the manufacturer's protocol and cultured in RPMI-1640 medium supplemented with 10% FBS and 1% P/S. All cell lines were grown at 37 °C in a 5% CO_2_ atmosphere.

### Isolation of exosomes from cell culture supernatants

PANC-1 cells (6.5 × 10^5^ cm^−2^) were incubated for 16 h in DMEM medium without FBS. Conditioned medium was collected and centrifuged for 20 min at 2,000*g* at 4 °C; the supernatant was transferred and centrifuged 30 min at 10,000*g* at 4 °C to remove cellular debris. The supernatant was then transferred to ultracentrifuge tubes and centrifuged for 60 min at 100,000*g* at 4 °C; the obtained pellet was washed in PBS and centrifuged for 60 min at 100,000*g* at 4 °C and used to determine exosomes protein content. Supernatants were precipitated using acetone and used to determine protein contents in supernatants depleted of vesicles.

### Measurement of NO_2_^−^ in supernatants

NO_2_^−^ amounts were measured by Griess reaction. Briefly, 100 μl of cell culture medium were mixed with 100 μl of Griess reagent and incubated at room temperature for 10 min. Then, the absorbance at 550 nm was measured in a Titertek microplate reader (Dasit, Cornaredo, Milan, Italy). The amount of NO_2_^−^ (as μM) in the samples was calculated from a sodium nitrite standard curve.

### Antibodies

Anti-BAG3 rabbit pAb (polyclonal Antibody) and murine mAbs (monoclonal Antibodies) and their F(ab')_2_ fragments were obtained from BIOUNIVERSA s.r.l., SA, Italy. The rabbit polyclonal anti-BAG3 was raised against the full-length BAG3 recombinant protein and used for western blotting at a 1:10,000 dilution. The anti-BAG3 murine mAb used in *in vitro* and *in vivo* studies specifically interacts with a portion of BAG3 protein (from aa 385 to aa 399) not overlapping the BAG domain. This was produced in endotoxin-free conditions by Nanotools (Teningen, DE), as well as control unrelated murine IgGs. The anti-BAG3 monoclonal murine clone AC-1 used for stainings specifically interacts with the BAG3 region from aa 18 to aa 33. Anti-GAPDH (6C5) antibody (sc-32233, 1:5,000), anti-phospho-p38 antibody (sc-17852-R, 1:1,000), anti-calnexin (H-70) antibody (sc-11398, 1:1,000), anti-Rab-4a (D-20) antibody (sc-312, 1:1,000) and anti-β-integrin-PE antibody (sc-13590, 1:200) were provided by Santa Cruz Biotechnology (Santa Cruz, CA, USA). Anti-phospho-Akt antibody (9271, 1:1,000) was provided by Cell Signaling (Boston, MA, USA). Anti-iNOS (610431, 1:5,000) and anti-COX-2 (610203, 1:5,000) antibodies were provided by BD Transduction Laboratories (San Diego, CA, USA). Human IgGs (I2511, 320 μg ml^−1^) and mouse IgG1s (ABIN125733, 320 μg ml^−1^) were provided by Sigma-Aldrich (St Louis, MO, USA) and Gmbh (Aachen, Germany), respectively. The anti-IL-6 receptor mAb Tocilizumab (20 μg ml^−1^) was purchased from Hoffmann-La Roche (Germany). The anti-IFITM-2 antibody (12769-1-AP, 1:1,000) was purchased from Proteintech (Chicago, IL, USA). DyLight 488-conjugated anti-rabbit IgG antibodies (1:500), 594-conjugated goat anti-mouse antibodies (1:500), mouse IgG F(ab')_2_ fragment (015-000-006, 320 μg ml^−1^) and peroxidase-conjugated secondary antibodies (1:5,000) were purchased from Jackson immunoresearch Laboratories (Baltimore, PA, USA).

### Chemicals and reagents

MTT ([3-(4,5-dimethylthiazol-2-yl)-2,5-diphenyl tetrazolium bromide]) (M2128), LPS (LPS from *Escherichia Coli* 0111:B4) (L4391), Polymyxin B sulphate (P0972), FluoroTag FITC conjugation kit (FITC1-1KT) and BSA (albumin from bovine serum) (A9418) were purchased from Sigma-Aldrich. Mouse IL-6 ELISA (88-7064-88) and Human IL-6 ELISA (88-7066-88) kits were provided by eBioscience (San Diego, CA, USA). Recombinant (r) BAG3 was produced as referenced in ref. 48. The PI3K inhibitor (PI3Ki) LY294002 and the p38 inhibitor (MAPKi) SB203580 were purchased from Calbiochem (Darmstadt, Germany). Monocyte Isolation Kit II (130-091-153) was purchased from Miltenyi Biotec S.r.l. (Bergisch Gladbach, Germany). Lymphocyte Separation Medium was obtained from Lonza.

### Cell transfections

Cells were transfected with IFITM-2-specific siRNA (si-IFITM-2) (Santa Cruz, CA, USA) or with a non-targeted (NT) siRNA (Santa Cruz, CA, USA) as a control, by using Transfectin (Bio-Rad, Hercules, CA, USA).

### Reverse transcription–PCR

RNA extraction was performed in phenol/chloroform (5:1) followed by overnight isopropanol precipitation. RNA was then pelleted by centrifugation at 15,000*g* for 30 min., washed two times with 70% ethanol and centrifuged at 15,000*g* for 30 min each time. Pellets were air-dried and resuspended in 30 μl of RNase-free water. One microgram of RNA was reverse-transcribed using the QuantiTect Reverse Transcription kit (205310, QIAGEN, Hilden, Germany) according to manufacturer's protocols. About 2 μl of the obtained complementary DNA were amplified in a total reaction volume of 20 μl. Complementary DNAs were analysed by PCR using HotStarTaq Master Mix (Qiagen), 0.6 μM primers (IL-6: forward (FW) 5′-CACGGCCTTCCCTACTTCAC-3′, reverse (RW) 5′-TGCAAGTGCATCATCGTTGT-3′; IL-10: FW 5′-TGATGCCCCAAGCTGAGAAC-3′, RW 5′-GCATTCTTCACCTGCTCCAC-3′; COX-2: FW 5′-ATCTACCCTCCTCAAGTCCC-3′, RW 5′-AACAACTGCTCATCACCCC-3′; iNOS: FW 5′-ATTCCCAGCCCAACAACAC-3′, RW 5′-TGAAAAATCTCTCCATTGCCC-3′) and RNase-free water. PCR products were detected by electrophoresis in 2% agarose gel (Sigma-Aldrich) and ethidium bromide staining. Uncropped blots and gels are shown in Supplementary Figs 7 and 8.

### Western blot

Cells were harvested and lysed in a buffer containing 20 mM HEPES (pH 7.5), 150 mM NaCl, 0.1% Triton (TNN buffer) supplemented with a protease inhibitors cocktail (Sigma) and subjected to three cycles of freezing–thawing. Lysates were then centrifuged for 20 min at 15,000*g* and stored at −80 °C. Protein amount was determined by Bradford assay (Bio-Rad, Hercules, CA) and 30 μg of total protein were separated on 10% SDS–PAGE gels and electrophoretically transferred to nitrocellulose membrane. Nitrocellulose blots were blocked with 10% nonfat dry milk in TBST buffer (20 mM Tris-HCl at pH 7.4, 500 mM NaCl and 0.01% Tween), and incubated with primary antibodies (used at 1:1,000 dilution) in TBST containing 5% nonfat dry milk overnight at 4 °C. Immunoreactivity was detected by sequential incubation with horseradish peroxidase-conjugated secondary antibodies (used at 1:5,000 dilution) and ECL detection reagents (Amersham Life Sciences Inc., Arlington Heights, IL, USA). Signal detection was performed using ImageQuant LAS 4000 (GE Healthcare, USA). Densitometry of bands was performed with ImageJ software (NIH, USA). The area under the curves, each relative to a band, was determined and the background was subtracted from the calculated values. Uncropped blots and gels are shown in Supplementary Figs 7 and 8.

### Animal studies

The research protocol was approved by the Ethics Committee of National Cancer Institute G. Pascale Foundation–IRCCS of Naples, in accordance with the institutional guidelines of the Italian Ministry of Health, protocol n. 49546. Informed consent for the use of human specimens was approved by the ‘S.S. Annunziata' Hospital Ethical Committee and obtained for all patients for this study. Sample size was chosen to ensure adequate and statistically significant results. Female CD1 nu/nu mice (6-week-old; Harlan Laboratories, Italy) were housed five per cage with food and water available *ad libitum* and maintained on a 12-h light/dark cycle under standard and specific pathogen-free conditions. Mice were acclimatized for 1 week before receiving injection of cancer cells. A total of 20 mice were used in this experiment and maintained in a barrier facility on HEPA-filtered racks. Animals were individually identified using numbered ear tags. All experiments were conducted in a biological laminar flow hood, and all surgical procedures were conducted with strict adherence to aseptic techniques. The mice were anesthetized using isoflurane. For injecting cancer cells, mice were prepped with 10% povidone-iodine. A longitudinal median laparotomy with a xipho-pubic incision was made, and the tail of the pancreas exteriorized gently. MIA PaCa-2 cells (1 × 10^6^) were suspended in 40 μl of PBS 1 × in a 1 ml syringe. Using a 25G needle, cells were injected into the tail of the pancreas and the injection point dubbed with sterile cotton. Once homeostasis was confirmed, the tail of the pancreas was returned into the abdomen and the wound was closed as a single layer using interrupted 5.0 silk sutures and skin staples. Three weeks after cell injection, tumour area was assessed using Vevo 2100 (Visualsonics, Canada) under anaesthesia. Mice were randomized into two groups in which tumour area average was ∼20 mm^2^: the control group received i.p. injection of 20 mg kg^−1^ of control mouse IgGs while the experimental group received 20 mg kg^−1^ of the murine anti-BAG3 mAb. Mice were treated every 48 h. At the end of the experiment animals were killed, and tumour and serum samples were collected for further analysis. For PDX model, fresh surgical human pancreatic carcinoma specimen was obtained from a patient who had undergone pancreatic resection at the Chieti University Hospital. Excess tumour tissue, not needed for clinical diagnosis was cut into 3–5 mm^3^ pieces in antibiotic-containing RPMI medium and then washed in cold sterile PBS. Pieces of non-necrotic tissue were selected and implanted in 7-week-old female NOD/ SCID mice into an s.c. pocket made by a small incision in the right flank of four animals. Tumour growth was monitored once per week by palpation. Once tumours reached a volume of about 500 mm^3^, explanted tumour specimens were cut and transplanted as described above into nu+/nu+ female mice to generate F0 cohort. The F0 tumours were then used to generate an F1 cohort, which was used for anti-BAG3 mAb studies. Initially, 15 mice were used for F_1_ tumour expansion. Mice were divided in two arms consisting of six mice each in which tumour volume average was ∼100 mm^3^. One group received i.p. injection every 48 h of 20 mg kg^−1^ anti-BAG3 mAb in PBS, whereas the other received PBS only (control group). Animals were weighted and tumour volume was measured by caliper twice weekly. Tumour volume is expressed in mm^3^ as calculating using the formula *D* × *d*^2^/2 were *D* is the longer diameter and *d* is the shorter diameter. At the end of the experiment animals were killed, and tumour and serum samples were collected for further analysis. For syngeneic model, mt4-2D murine cells (0.25 × 10^6^) were suspended in a solution 1:1 PBS 1 × /matrigel and injected into the right flank of female C57BL6 mice (6-week-old; Harlan Laboratories). After 10 days, mice were divided in 2 arms consisting of 10 mice each in which tumour volume average was ∼100 mm^3^. One group received i.p. injection every 48 h of 20 mg kg^−1^ anti-BAG3 mAb in PBS, whereas the other received unrelated IgGs for 4 weeks. Animals were weighted and tumour volume was measured by caliper twice weekly. Serum samples from Ikkalpha f/f mice were kindly provided by Prof. Karin M. lab. Normal pancreas sera were from mice on normal diet of age 18 months; chronic pancreatitis sera were from Pdk-cre; Ikkalpha f/f at same age and same diets of normal controls. PDAC serum samples were collected from Pdx-Cre; KrasG12D; Ikkalpha f/f at the age of 4 months when tumour was already very advanced.

### Immunoprecipitation from mice sera

Blood samples were collected from the right retroorbital plexus of anesthetized mice. For immunoprecipitation of BAG3 protein from mice sera the anti-BAG3 mAb AC-1 was coupled to Dynabeads (Invitrogen) following the manufacturer's instructions. About 60 μl of mouse sera were immunoprecipitated at 4 °C overnight and then analysed by western blot using a rabbit anti-BAG3 polyclonal primary antibody.

### Immunofluorescence

For paraffin-embedded sections, immunofluorescence protocol included deparaffination in Clear-Rite 3 (ThermoScientific, Waltham, MA, USA) rehydration through descending degrees of alcohol up to water, non-enzymatic antigen retrieval in sodium citrate buffer 10 mM, 0.05% Tween, pH 6.0, for 3 min in microwave at 700 W. After washing, non-specific binding was blocked with 1% FBS in PBS 1 × . Sections were then incubated with several primary antibodies: anti-F4/80 and anti-CD68 polyclonal antibodies obtained from Abcam (Cambridge, U.K. at 1:200) and anti-BAG3 monoclonal antibodies (BIOUNIVERSA s.r.l. at 1:240), overnight at 4 °C in a humidified chamber. After another washing step, sections were incubated with the secondary antibodies. Nuclei were counterstained with 1 μg ml^−1^ Hoechst 33342 (Molecular Probes, Oregon, USA). Negative controls were performed using all reagents except the primary antibody. For cell cultures, cells were cultured on coverslips in six-well plates to 60–70% confluence; after 16 h, coverslips were washed in 1 × PBS and fixed in 3.7% formaldehyde in PBS 1 × for 30 min at room temperature, and then incubated for 5 min with PBS 1 × /0.1 M glycine. After washing, coverslips were permeabilized with 0.1% Triton X-100 for 5 min, washed again and incubated with blocking solution (10% normal goat serum in PBS 1 × ) for 1 h at room temperature. Following incubation at 4 °C overnight with 3 μg ml^−1^ of RAB7a polyclonal antibody and 3 μg ml^−1^ of anti-BAG3 mouse monoclonal antibody AC-1, coverslips were washed three times with PBS 1 × . After incubation with secondary antibodies at room temperature for 45 min, coverslips were again washed three times in PBS and then in distilled water. The coverslips were then mounted on a slide with interspaces containing moviol. Samples were analysed using a confocal laser scanning microscope (Leica SP5, Leica Microsystems, Wetzlar, Germany). Images were acquired in sequential scan mode by using the same acquisitions parameters (laser intensities, gain photomultipliers, pinhole aperture, objective × 63, zoom 2) when comparing experimental and control material. For figure preparation, brightness and contrast of images were adjusted by taking care to leave a light cellular fluorescence background for visual appreciation of the lowest fluorescence intensity features and to help comparison among the different experimental groups. Final figures were assembled using Adobe Photoshop 7 and Adobe Illustrator 10. Leica Confocal Software and ImageJ were used for data analysis.

### Immunohistochemistry

Four-micron-thick sections of each tissue, mounted on poly-L-lysine-coated glass slides, were analysed by immunohistochemistry (IHC) using the anti-BAG3 mAb AC-2 (BIOUNIVERSA s.r.l.). IHC protocol included deparaffination in Clear-Rite 3, rehydration through descending degrees of alcohol up to water, incubation with 3% hydrogen peroxidase for 5 min to inactivate endogenous peroxidases, non-enzymatic antigen retrieval in EDTA at pH 8.0 for 30 min at 95 °C. After rinsing with phosphate-buffered saline (PBS 1 × ), samples were blocked with 5% foetal bovine serum in 0.1% PBS/BSA and then incubated for 1 h at room temperature with the mAb in saturating conditions. The standard streptavidin–biotin linked horseradish peroxidase technique was then performed, and 3,3′-diaminobenzidine was used as a substrate chromogen solution for the development of peroxidase activity. Finally, the sections were counterstained with haematoxylin; slides were then coverslipped using a synthetic mounting medium.

### Cytokines determination

Five tumours from anti-BAG3 mAb-treated mice and six tumours from control IgG-treated mice were collected, weighed and added to 9 × volume of lysis buffer (50 mM Tris-HCL with 2 mM EDTA, pH 7.4) to which protease and phosphatase inhibitors were added. Tissues were processed by a Potter-Elvehjem homogenizer. Following homogenization, lysates were centrifuged for 2 min in a microfuge at 13,000*g*. Each sample was analysed using Myriad RBM Mouse Inflammation MAP v. 1.0 array (Austin, TX, USA).

### Identification of IFITM-2 as receptor for BAG3

Murine J774.A1 macrophages were incubated with His-tagged recombinant rBAG3, mechanically lysed and subjected to membrane fractionation. Plasma membrane proteins were natively solubilized using 1% CALX173ACE Calixar molecule and His-tagged rBAG3 containing complexes were pulled down by affinity chromatography. Protein sample was precipitated by 20% trichloroacetic acid and resuspended with 25 mM (NH4)HCO_3_, 2% sodium deoxycholate, pH 8.0, prior to the trypsic digestion. After sodium deoxycholate removal by trifluoroacetic acid precipitation, samples were analysed by LC–MS/MS, identifying IFITM-2 as a potential receptor for BAG3. In detail, digested samples were preconcentrated using an Acclaim PepMap100 C18 capillary-column (5 μm, 100 Å, 300 μm × 5 cm, Dionex, Courtaboeuf, France) with 98/2 H_2_O/ACN, 0.05% TFA buffer. Peptides separation were then carried out using an Acclaim PepMap100 C18 nano-column (3 μm, 100 Å, 75 μm × 15 cm, Dionex) with 10/80 H_2_O/ACN, 0.1% formic acid buffer. MS/MS were performed with a LTQ Velos linear ion trap mass spectrometer (Thermo Scientific). Data were analysed with ProteomeDiscoverer 1.1 software (MASCOT algorithm, v2.2.4) using the SwissProt database (UniprotKB, v12/2012) with the following criteria: enzyme: trypsin; max missed cleavage: 2; FDR: 0.01 and 0.05; precursor mass tolerance: 0,4 Da; fragment mass tolerance: 0,4 Da; dynamic modification: oxidation (M) et deamidated (NQ); Static modification: carbamidomethyl (C).

J774.A1 macrophages not incubated with rBAG3 served as negative control. To validate IFITM-2 as a receptor of BAG3, murine J774.A1 macrophages were incubated with His-tagged recombinant rBAG3. After mechanical cell lysis and membrane fractionation by sequential centrifugations, plasma membrane complexes were natively extracted with 1% CALX173ACE Calixar molecule. Partners interacting with IFITM-2 were co-immunoprecipitated by immuno-affinity chromatography using an anti-IFITM-2 agarose resin. Interactions of IFITM-2 with rBAG3 were monitored by western blot. J774.A1 macrophages not incubated with rBAG3 served as a negative control.

### Statistical analysis

Results are expressed as mean±s.d. or ±s.e.m. Data were analysed by Student's *t*-test using MedCalc statistical software version 13.3.3 (Ostend, Belgium). *P* values from 0.01 to 0.05, from 0.001 to 0.01 or<0.001 were considered significant (*), very significant (**) or highly significant (***), respectively.

## Additional information

**How to cite this article:** Rosati, A. *et al.* BAG3 promotes pancreatic ductal adenocarcinoma growth by activating stromal macrophages. *Nat. Commun.* 6:8695 doi: 10.1038/ncomms9695 (2015).

## Supplementary Material

Supplementary Information Supplementary Figures 1-8 and Supplementary Table 1

## Figures and Tables

**Table 1:** IL-6 contents in LPS and BAG3-stimulated monocytes.

	**IL-6 (ng ml** ^**−1**^ **)**			
	**Control**	**LPS (10 ng ml** ^**−1**^ **)**	**LPS (100 ng ml** ^**−1**^ **)**	**rBAG3 (6 μg ml** ^**−1**^ **)**
Donor 1	1.6	2.8	3.8	3.6
Donor 2	2.0	2.6	5.0	5.4
Donor 3	10.5	12.5	12.6	17.0
Donor 4	6.9	7.8	5.9	9.0
Donor 5	4.7	8.2	8.3	8.1

IL-6, interleukin-6; LPS, lipopolysaccharide; BAG3, recombinant *Bcl-2-*associated athanogene 3.

**Table 2:** Percentage of PDAC cell growth inhibition by Tocilizumab.

	**% of cell growth inhibition (±s.d.)** *	
	**Tocilizumab**	**Human IgG**
rIL-6	82±6.2	7±2.4
rBAG3 stimulated donor 4	22±6.2	0±0.0
rBAG3 stimulated donor 5	9±0.8	0±0.0

PDAC, pancreatic ductal adenocarcinoma; rBAG3, recombinant *Bcl-2-*associated athanogene 3; rIL-6, recombinant interleukin-6.

^*^Data are from duplicate samples.

**Table 3:** Peritoneal metastasis inhibition by anti-BAG3 mAb.

	**Animals without peritoneal metastasis**	**Animals with peritoneal metastasis**
Control	4	5
Anti-BAG3 mAb	7	0

BAG3, recombinant *Bcl-2-*associated athanogene 3; mAb, monoclonal antibody.

*P*=0.03 calculated with Fisher's exact test for 2 × 2 contigency table.
