# Supplementary material for: BAG3 promotes pancreatic ductal adenocarcinoma growth by activating stromal macrophages
Source: Nat Commun. 2015 Nov 2;6:8695. doi: 10.1038/ncomms9695 (PMC4659838; doi:10.1038/ncomms9695)
Supplement: Supplementary Information — Supplementary Figures 1-8 and Supplementary Table 1 [file ncomms9695-s1.pdf]

Supplementary Figure 1 - Characterization of rBAG3 binding on macrophages cell surface.

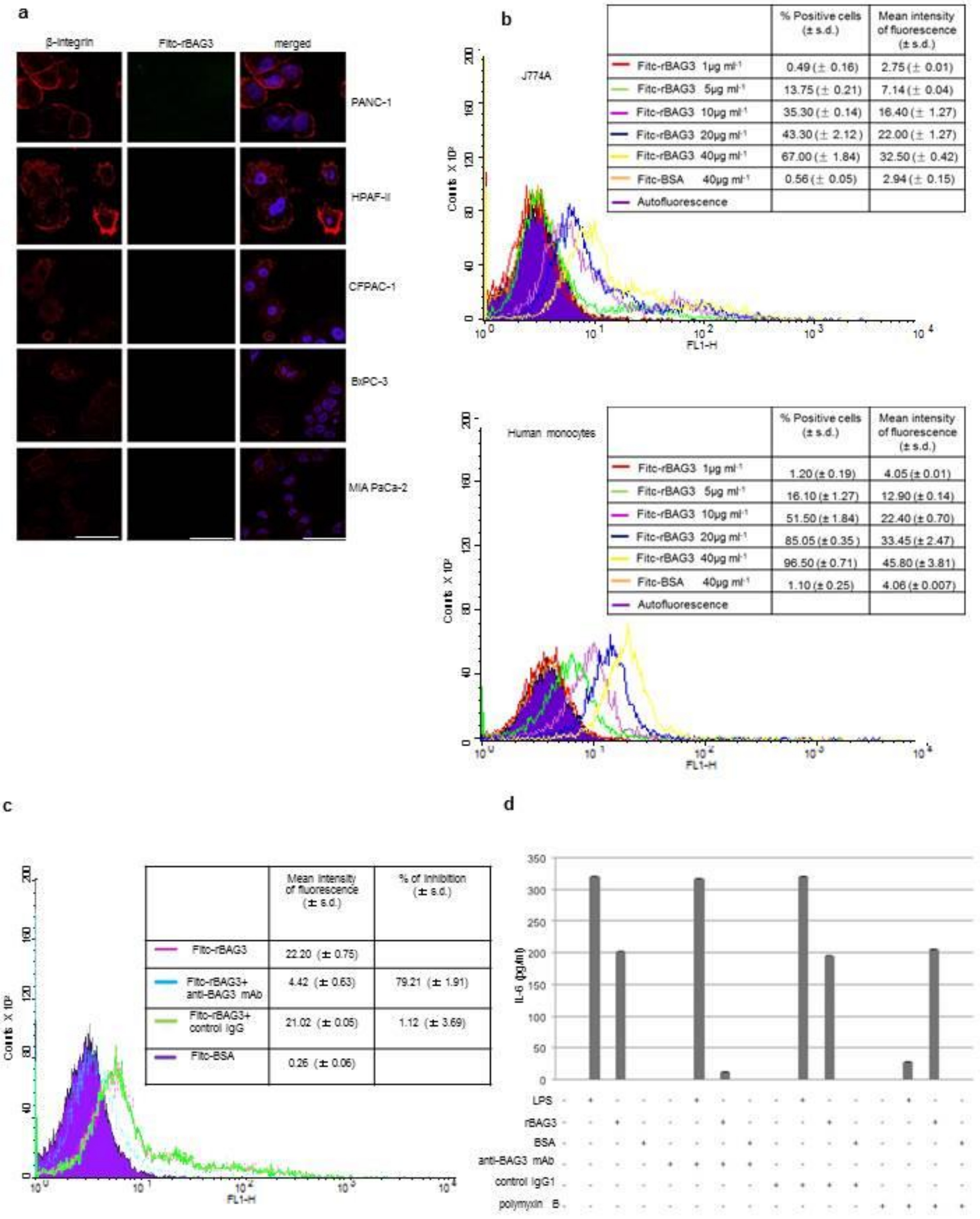

J774A	% Positive cells (± s.d.)	Mean intensity of fluorescence (± s.d.)
Fitc-rBAG3 1μg ml⁻¹	0.49 (± 0.16)	2.75 (± 0.01)
Fitc-rBAG3 5μg ml⁻¹	13.75 (± 0.21)	7.14 (± 0.04)
Fitc-rBAG3 10μg ml⁻¹	35.30 (± 0.14)	16.40 (± 1.27)
Fitc-rBAG3 20μg ml⁻¹	43.30 (± 2.12)	22.00 (± 1.27)
Fitc-rBAG3 40μg ml⁻¹	67.00 (± 1.84)	32.50 (± 0.42)
Fitc-BSA 40μg ml⁻¹	0.56 (± 0.05)	2.94 (± 0.15)
Autofluorescence		

Human monocytes	% Positive cells (± s.d.)	Mean intensity of fluorescence (± s.d.)
Fitc-rBAG3 1μg ml⁻¹	1.20 (± 0.19)	4.05 (± 0.01)
Fitc-rBAG3 5μg ml⁻¹	16.10 (± 1.27)	12.90 (± 0.14)
Fitc-rBAG3 10μg ml⁻¹	51.50 (± 1.84)	22.40 (± 0.70)
Fitc-rBAG3 20μg ml⁻¹	85.05 (± 0.35)	33.45 (± 2.47)
Fitc-rBAG3 40μg ml⁻¹	96.50 (± 0.71)	45.80 (± 3.81)
Fitc-BSA 40μg ml⁻¹	1.10 (± 0.25)	4.06 (± 0.007)
Autofluorescence		

	Mean intensity of fluorescence (± s.d.)	% of inhibition (± s.d.)
Fitc-rBAG3	22.20 (± 0.75)	
Fitc-rBAG3+ anti-BAG3 mAb	4.42 (± 0.63)	79.21 (± 1.91)
Fitc-rBAG3+ control IgG	21.02 (± 0.05)	1.12 (± 3.69)
Fitc-BSA	0.26 (± 0.06)	

(a) Human PDAC cell lines were treated as indicated in Figure 1 panel F. Cells were analyzed for FITC-rBAG3 binding by a Zeiss LSM confocal microscope (scale bar: 50 μm). **(b)** J774A.1 and human monocytes were stained with different concentrations of FITC-rBAG3 protein, or FITC-BSA as a control, and analyzed by flow cytometry. Data are from duplicate samples and repeated twice. Error bars indicate s.d. **(c)** J774 A.1 cells were incubated with FITC-rBAG3 protein (14 nM) alone or in presence of an anti-BAG3 monoclonal antibody (420 nM) or murine IgG1 (420 nM), in PBS containing 2% FBS/0.1% NaN_3, for 30 minutes on ice. After incubation, cells were washed with PBS and analyzed by flow cytometry. Data are from triplicate samples and confirmed in three separate experiments . Error bars indicate s.d. **(d)** IL-6 was measured in supernatant of J774 A.1 cells treated with LPS (10 ng ml^{-1}), rBAG3 (8 µg ml^{-1}) or BSA (8 µg ml^{-1}) for 24 hours in absence or presence of a monoclonal anti-BAG3 antibody (320 µg/ml) or mouse IgG1 (320 µg ml^{-1}) or polymyxin B (5 µg ml^{-1}). After treatment, cell supernatants were collected and analyzed with a mouse ELISA IL-6 Kit. Data are from duplicate samples and repeated three times. Error bars indicate s.d.

Supplementary Figure 2- anti-BAG3 mAb has no direct effect on PDAC cell lines viability.

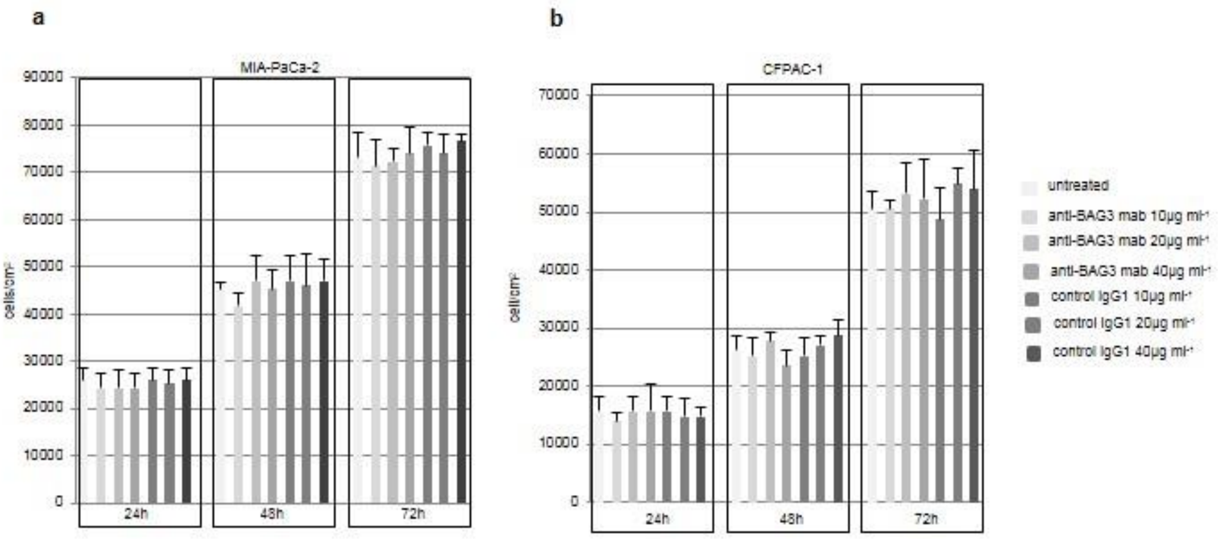

(a) MIA PaCa-2 cells were cultured for the indicated times with increasing concentrations of anti-BAG3 mAb or control IgG1. Cells were harvested, stained with trypan blue and counted. Cell mortality was no more than 5% in all samples. Data are from duplicate samples and repeated two times. Error bars indicate s.d. **(b)** CFPAC-1 cells were treated and analyzed as described above.

Supplementary Figure 3- Identification of IFITM-2 as potential receptor for BAG3.

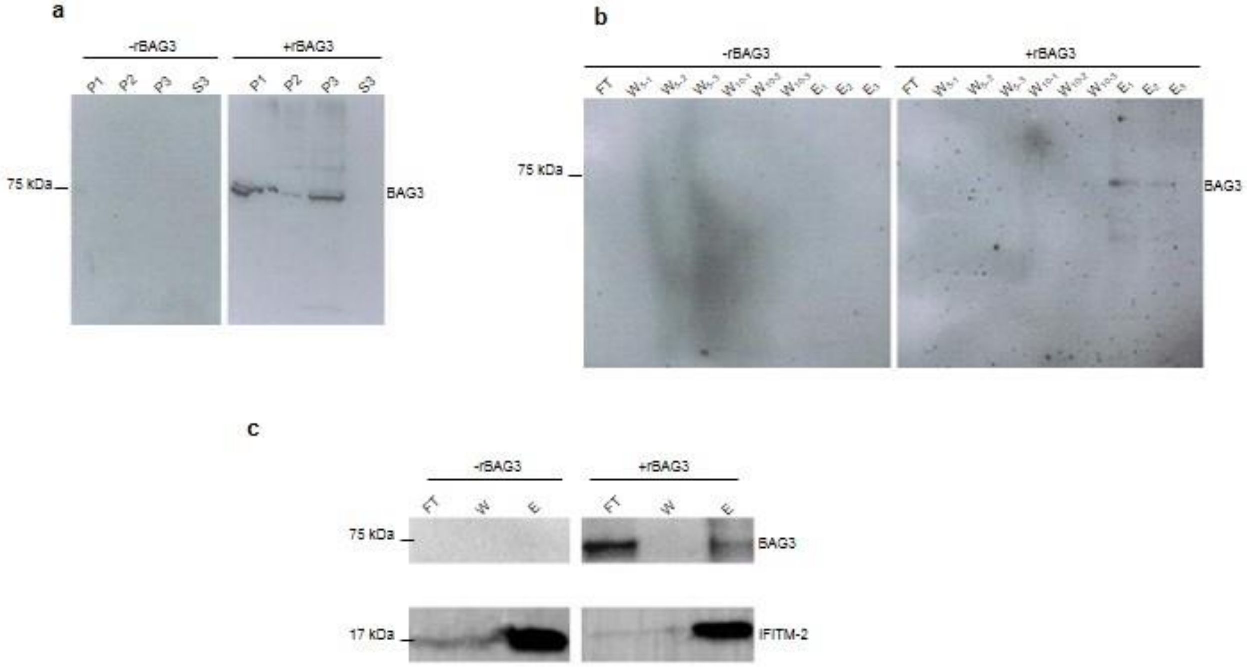

(a) Cells were incubated (+rBAG3) or not (no BAG3) with His-tagged rBAG3 protein, then washed 3 times in PBS and mechanically lysed. Membrane fractions were obtained by sequential centrifugations after lysis. P1, pellet after centrifugation at 500 g (cell debris, whole cells); P2, pellet after centrifugation at 15,000 g (ER, mitochondria, nucleus); P3, pellet after centrifugation at 100,000 g (plasma membranes); and S3, supernatant after centrifugation at 100,000 g. All fractions (P; P2; P3 and S3) were analyzed by western blotting using an anti-His-HRP antibody that recognizes the His-tagged BAG3 recombinant protein. **(b)** Purification of rBAG3- containing complexes from solubilized plasma membranes (P3 fraction) prepared from J774.A1 cells incubated or not with His-tagged rBAG3. In the flow-through (FT) as well as in the wash fractions (W5-1, W5-2, W5-3, W10-1, W10-2, W10-3), no rBAG3 was recovered. rBAG3 was eluted in the two first elution fractions, E1 and E2. **C)** Plasma membranes from J774A.1 cells, incubated (+rBAG3) or not (no BAG3) with rBAG3 protein, were prepared and solubilized. Soluble fraction was incubated with an anti-IFITM-2 agarose resin. After washing the resin, specific bound proteins were eluted, separated by SDS-PAGE, and transferred to PDVF membrane. Immune-detection was performed

using an anti-His horseradish peroxidase- conjugated antibody and anti-human IFITM-2 primary antibody followed by a goat anti-rabbit IgG horseradish peroxidase- conjugated secondary antibody. FT, flowthrough; W1, wash 1; E, elution. Experiments described in this figure were made in collaboration with Calixar (Lyon, France).

Supplementary Figure 4- BAG3R480, a mutant that lost its ability to interact with Hsp70, is still capable of binding and activating monocytes/macrophages.

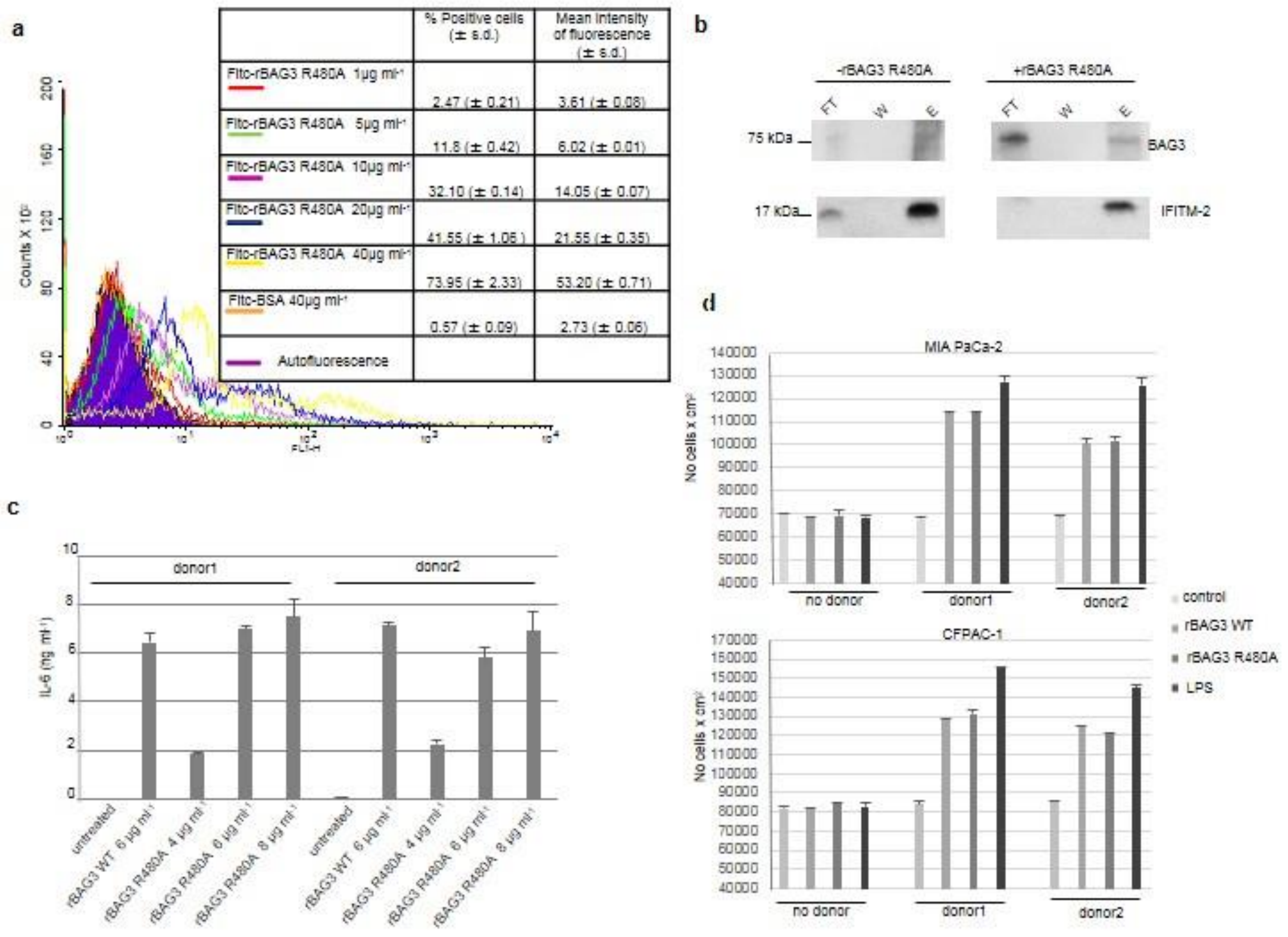

Site- directed mutagenesis was performed on BAG3 full-lenght coding sequence cloned in pET30a(+) plasmid vector (Novagen, Podenzano, Italy), using the QuickChange Site-Directed Mutagenesis kit (Agilent, Santa Clara, CA, US) according to the supplier's protocol. Primers used to create the mutated sequence encoding the R480A amminoacidic substitution are listed below. Arginine 480 is underlined and altered nucleotides are in bold: BAG3wt 5'-GAT GTG CGT CAG GCC <u>AGG</u> AGA GAC GGT GTC AG-3'; BAG3-R480A 5'-GAT GTG CGT CAG GCC **<u>GCG</u>** AGA GAC GGT GTC AG-3'. Mutated plasmids were isolated and checked by sequencing before trasformation in E.coli BL21(DE3)pLysE (Invitrogen, #C6565-03). The protein produced in bacteria was affinity purified on HIS-Select Nickel Affinity Gel (Sigma-Aldrich, #P6611) according to the manifacturer's protocol, and the mutation was verified by MS analysis.

(a) J774A.1 cells were stained with different concentrations of FITC-rBAG3R480 protein, or FITC-BSA as a control, and analyzed by flow cytometry. Data are from triplicate samples and repeated three times. Error bars indicate s.d. **(b)** Plasma membranes from J774A.1 cells, incubated (+rBAG3R480A) or not with His-tagged rBAG3R480A, were solubilized and incubated with an anti-IFITM-2 agarose resin. Immune-precipitates were analyzed as described in Figure S3 panel D; FT, flow-through; W1, wash 1; E, elution. **(c)** Isolated human monocytes (>98% $CD14^{+}$) were treated with rBAG3WT or with rBAG3R480A at the indicated concentrations for 16 hours. After treatment, supernatants were collected and analyzed with human IL-6 ELISA. IL-6 concentrations were evaluated by comparing the OD values of the samples with those of a recombinant IL-6 standard curve. rBAG3R480A induces IL-6 production by monocytes to the same extent of rBAG3WT. Data are from quadruplicate samples obtained in two independent experiment. Error bars indicate s.d. **(d)** MIA PaCa-2 (**upper panel)** and CFPAC-1 (**lower panel)** cells were cultured in DMEM supplemented with 0.15% FBS (no donor) or conditioned medium from monocytes treated with LPS (100 ng ml^{-1}), rBAG3WT (6 µg ml^{-1}) or rBAG3R480A (6 µg ml^{-1}) for 16 hours. After 72 hours of incubation, cells were analyzed by MTT assay. Supernatant from rBAG3R480A-treated monocytes promotes PDAC cell lines growth to the same extent of supernatants from monocytes treated with rBAG3WT. Data are from duplicate samples. Error bars indicate s.d.

Supplementary Figure 5- BAG3 ability to activate monocytes/macrophages does not depend on binding to Hsp70.

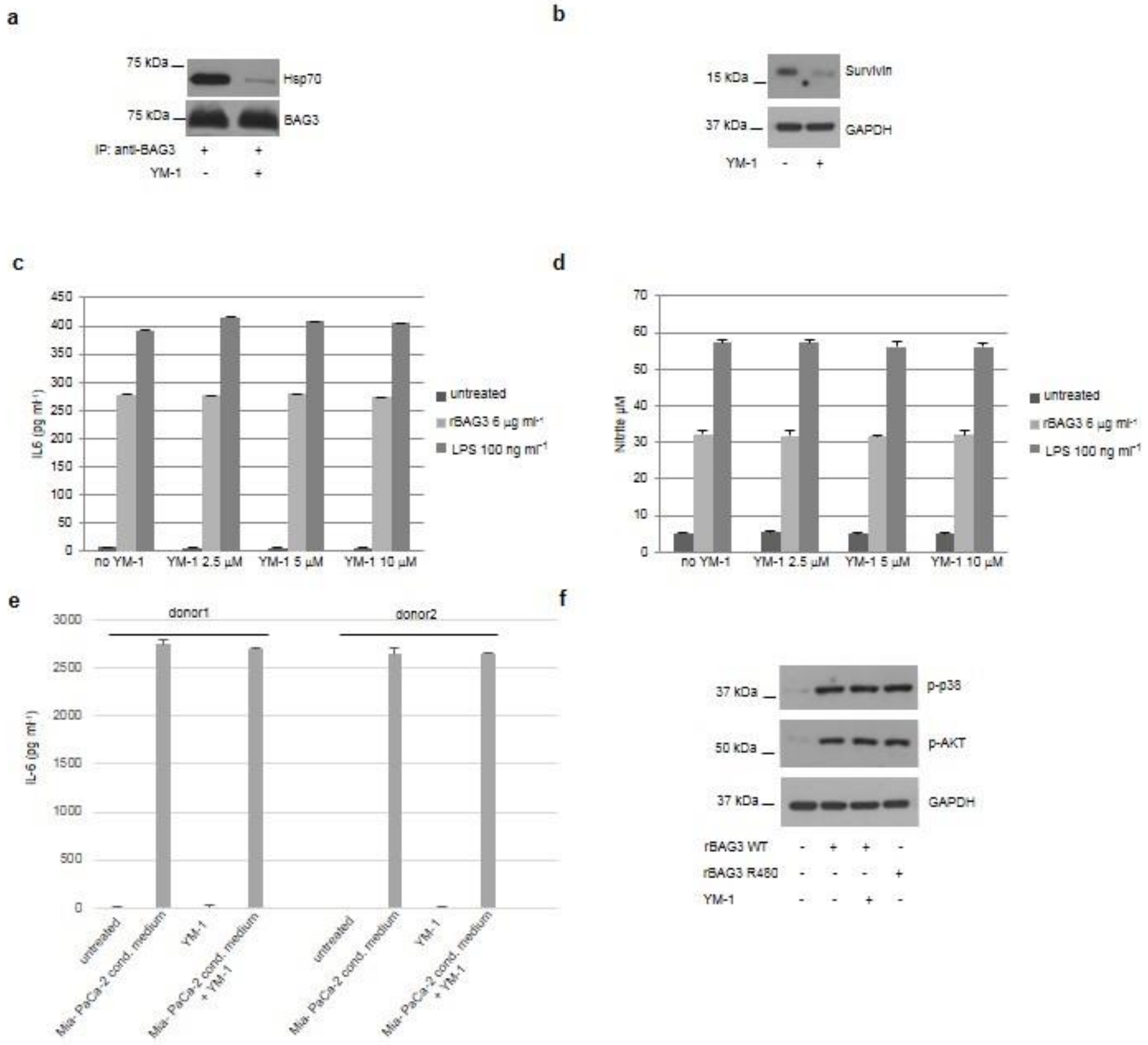

(a) J774.A1 protein extracts were immune-precipitated with an anti-BAG3 mAb (rabbit polyclonal anti-BAG3) in presence or absence of YM-1 inhibitor (purchased from Vincibiochem, Vinci, Italy). Immunoblot was performed to detect the quote of Hsp70 interacting protein using the anti-Hsp-70 antibody (mouse monoclonal sc-7298 obtained from Santa Cruz Biotechnology at 1:5,000) and an anti-BAG3 antibody (rabbit polyclonal anti-BAG3). **(b)** J774.A1 cells were cultured in the presence or absence of the YM-1 inhibitor (10 μM) for 4 hours. Survivin levels were evaluated by WB using an anti-survivin antibody (mouse monoclonal sc-17779 obtained from Santa Cruz Biotechnology at

1:1,000) and an anti-GAPDH was used as loading control. These data confirm the ability of the inhibitor YM-1 to block the interaction of BAG3 with Hsp70 and the induction of survivin[33] in our experimental models. **(c)** J774A.1 cells were incubated with LPS (100 ng ml^{-1}) or rBAG3 (6 µg ml^{-1}) in absence or presence of YM1 inhibitor at the indicated concentrations. After treatment, cell culture supernatants were analyzed with a mouse ELISA IL-6 Kit. Data are from duplicate samples and repeated two times. Error bars indicate s.d. **(d)** Cells were treated as described above and nitrite release was measured. These data demonstrate that inhibiting the interaction between BAG3 and Hsp70 does not affect the ability of BAG3 to activate monocytes. Data are from duplicate samples and confirmed in three separate experiments. Error bars indicate s.d. **(e)** Isolated human monocytes (>98% $CD14^{+}$) were cultured in RPMI supplemented with 0.15% FBS (untreated) or in MIA PaCa-2- conditioned medium, treated or not for 16 hours with YM-1 inhibitor (10 µM). YM-1 inhibitor (10 µM) alone was also added to the culture as a further control. After treatment cell culture supernatants were analyzed with a human IL-6 ELISA Kit**.** MIA PaCa-2- conditioned medium was fully capable of activating monocytes despite the presence of the YM-1 inhibitor. Data are from duplicate samples. Error bars indicate s.d. **(f)** J774.A1 cells were treated with rBAG3WT in the presence or not of the YM1 inhibitor (10 µM) for 4 hours or with rBAG3R480 (6 µg ml^{-1}). Cell extracts were analyzed by western blotting using anti-phospho-AKT and anti-phospho-p38 polyclonal antibodies; anti-GAPDH was used as loading control. These results show that the use of an inhibitor of Hsp70 binding or of a mutant that cannot bind Hsp70 does not impair the ability of BAG3 of activating the p38/Akt signaling pathways in macrophages.

Supplementary Figure 6- anti-BAG3 mAb reduces tumor growth and while cross-reacting with murine BAG3 shows no major toxic effects.

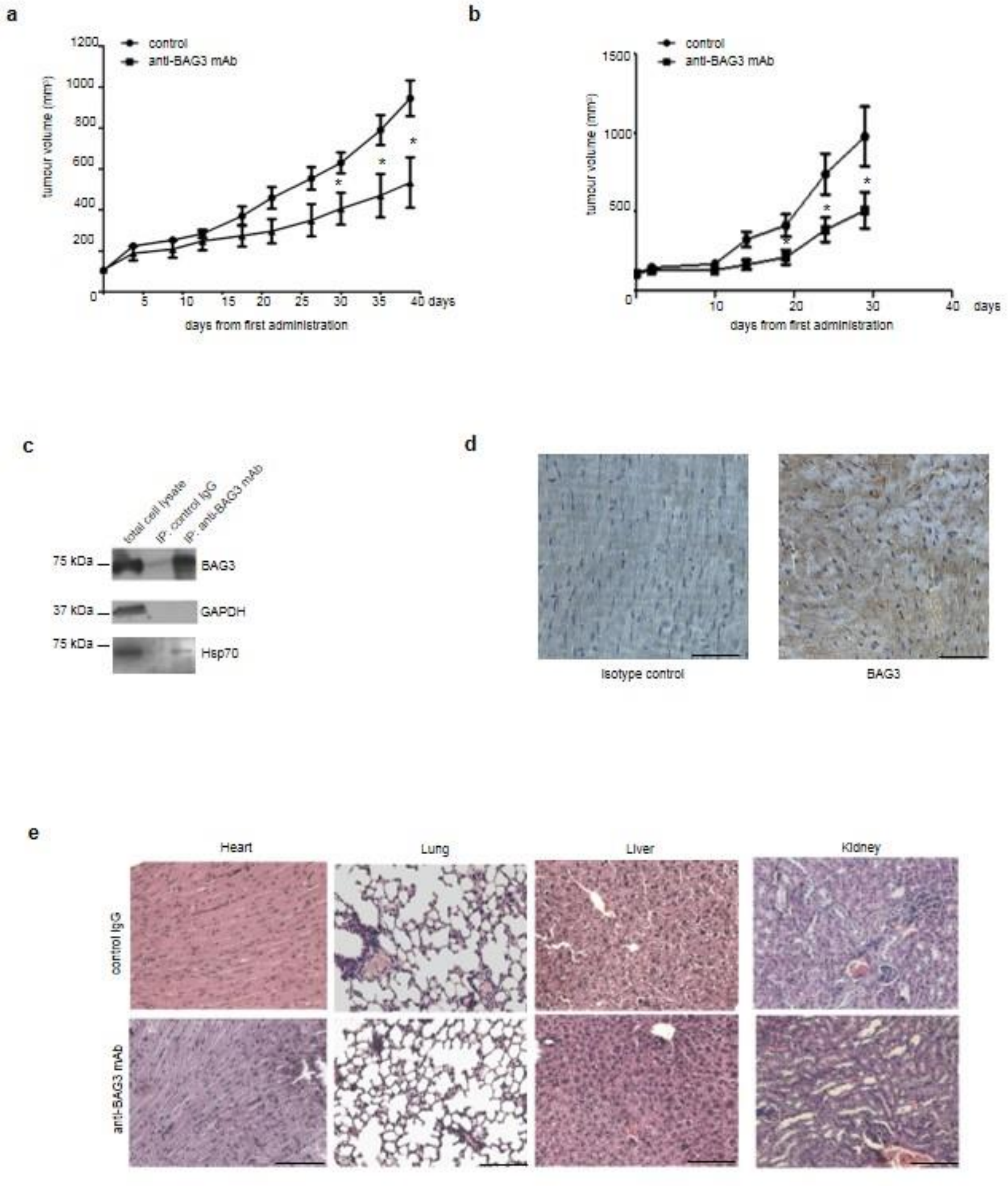

(a) *In vivo* response of PDX tumor to treatment with vehicle (PBS) or anti-BAG3 mAb (same experiment as in 4A). Results are here expressed as mean tumor volume +/- s.e.m. **(b)** *In vivo* response of syngeneic tumor to treatment with vehicle (PBS) or anti-BAG3 mAb (same experiment as in 4B). Results are expressed as mean tumor volume +/- s.e.m. **(c)** β–TC-6 (murine pancreatic

insulinoma cells) were grown in DMEM supplemented with 15% FBS. For immune-precipitation assays, cells were lysed in HNT buffer (Hepes 20 mM pH 7.5, NaCl 150 mM, Triton 0.1%) supplemented with a protease inhibitor cocktail (Sigma) on ice for 20 minutes. After 5 cycles of freeze and thaw, cell membranes were centrifuged at 10,000 x g. 500 μg of soluble proteins were subjected to immune-precipitation with 5 μg of anti-BAG3 mAb or 5 μg of control IgG. Antibodies were coupled to DynaBeads (Life Technologies) and all subsequent steps were performed following manufacturer's instructions. Immune-precipitates were then loaded on SDS-PAGE and analyzed for presence of BAG3 and Hsp70 by Western blotting and GAPDH as negative quality control. **(d)** Heart tissues from control nude mice were embedded in paraffin and analyzed by IHC (see experimental procedures) for the anti-BAG3 mAb, antigen retrieval was performed with EDTA at pH 8.0, non-specific IgG were used as negative control (scale bar 50 μm) **(e)** Specimens from mice treated with control IgG or anti-BAG3 mAb were analyzed by H&E staining (scale bar: 100 μm).

Supplementary Figure 7- uncropped blots and gels 1

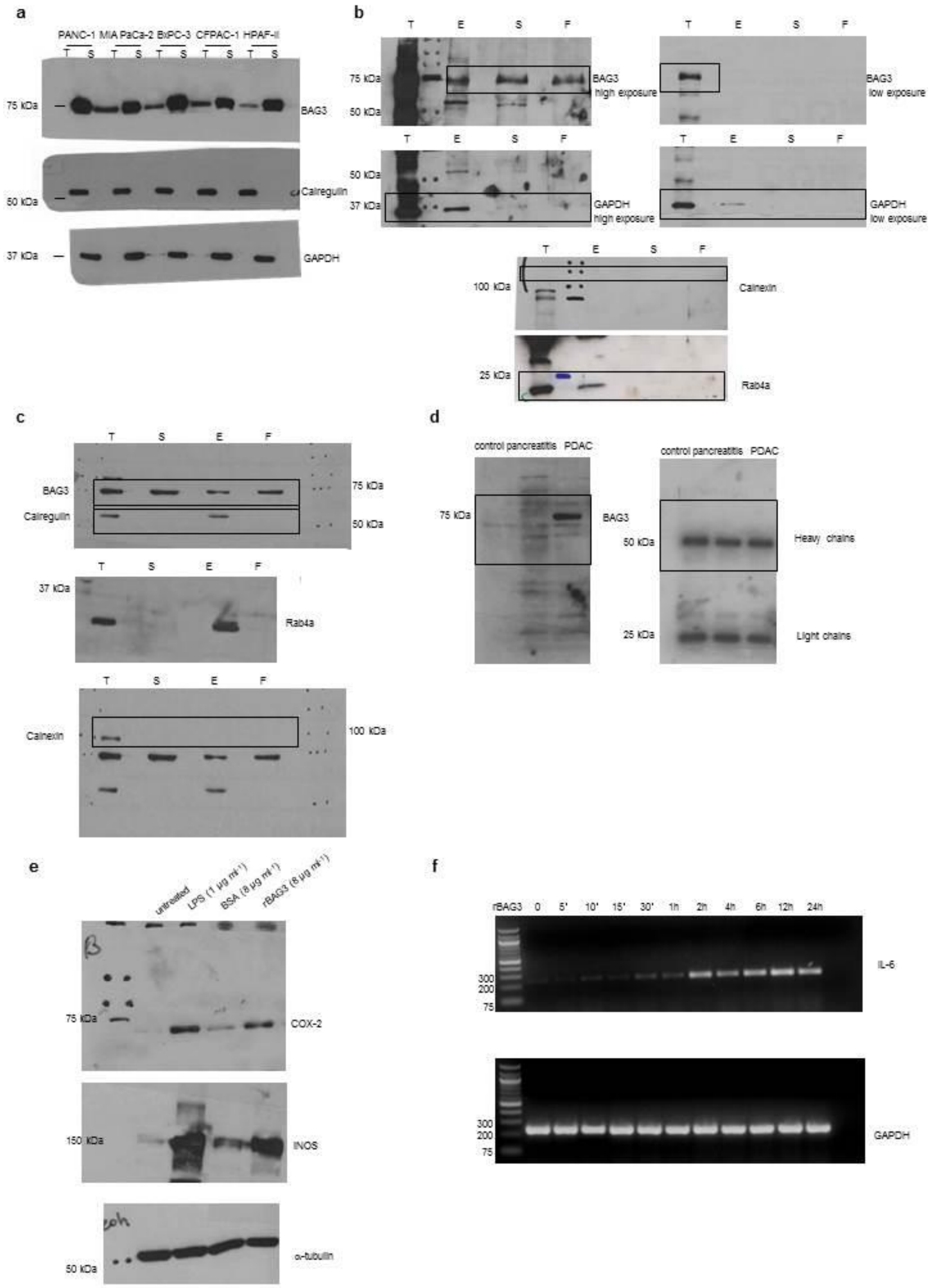
a
PANC-1 MIA PaCa-2 BxPC-3 CFPAC-1 HPAF-II
T S T S T S T S T S
75 kDa
BAG3
50 kDa
Calregulin
37 kDa
GAPDH
b
T E S F
75 kDa
50 kDa
BAG3 high exposure
BAG3 low exposure
50 kDa
37 kDa
GAPDH high exposure
GAPDH low exposure
100 kDa
Calnexin
25 kDa
Rab4a
c
T S E F
BAG3
Calregulin
75 kDa
50 kDa
37 kDa
Rab4a
Calnexin
100 kDa
d
control pancreatitis PDAC
75 kDa
BAG3
50 kDa
Heavy chains
25 kDa
Light chains
e
untreated
LPS (1 μg ml⁻¹)
BSA (8 μg ml⁻¹)
rBAG3 (8 μg ml⁻¹)
75 kDa
COX-2
150 kDa
iNOS
50 kDa
α-tubulin
f
rBAG3 0 5' 10' 15' 30' 1h 2h 4h 6h 12h 24h
300
200
75
IL-6
GAPDH

(a) blots from Figure 1 panel A. **(b)** blots from Figure 1 panel B:PANC-1. **(c)** blots from Figure 1 panel B: MIA PaCa-2. **(d)** blots from Figure 1 panel E. **(e)** blots from Figure 1 panel H. **(f)** gels from Figure 1 panel I. Black boxes indicates areas used in the main display items.

Supplementary Figure 8- uncropped blots and gels 2

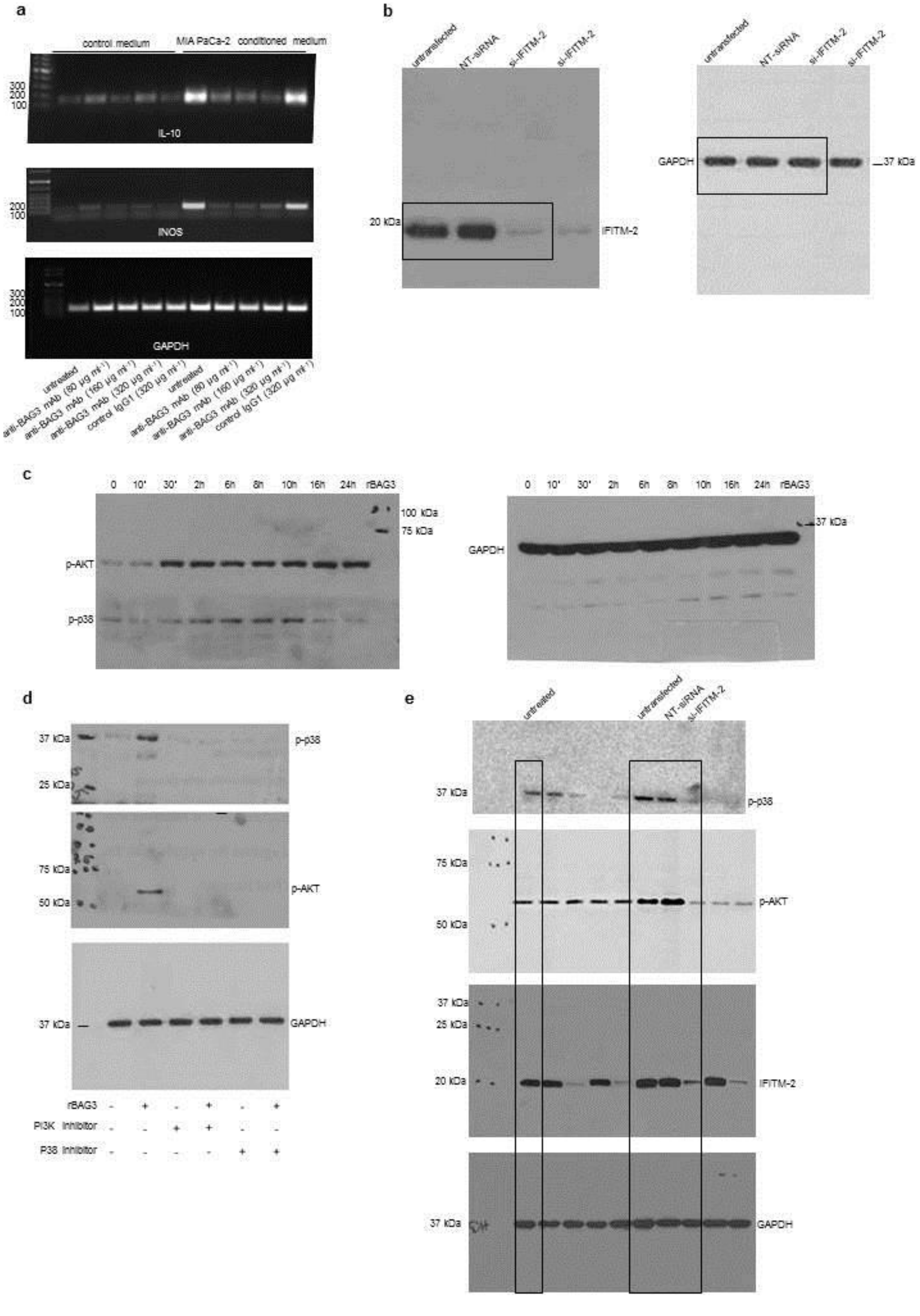

(a) gels from Figure 2 panel C. **(b)** blots from Figure 3 panel A. **(c)** blots from Figure 3 panel D. **(d)** blots from Figure 3 panel E. **(e)** blots from Figure 3 panel G. Black boxes indicates areas used in the main display items.

Supplementary Table 1. LC-MSMS and sequence analyses on proteins present only in +rBAG3 sample.

**Protein identification**	**Sequence**
IFITM-2	MVGDVVGAQAYASTAK
	KMVGDVVGAQAYASTAK
	TTVINMPR

IFITM-2 peptides identified are reported
http://figshare.com/s/86bc685c57be11e5868406ec4b8d1f61
